# Ossified Superficial Infrapatellar Bursa: A Case Report

**DOI:** 10.7759/cureus.35500

**Published:** 2023-02-26

**Authors:** Sai Krishna MLV, Aparna Kadiveti, Ravi Mittal

**Affiliations:** 1 Orthopaedics, All India Institute of Medical Sciences, New Delhi, IND; 2 Orthodontics and Dentofacial Orthopaedics, Narayana Dental College, Nellore, IND

**Keywords:** infrapatellar bursa, knee, bursitis, calcification, ossification

## Abstract

Bursitis, though treatable conservatively, very rarely can have ossification and calcification in its substance which requires surgical intervention. The patient should be investigated for any coexisting metabolic bone disorders before proceeding with surgical intervention. The excision biopsy of such a specimen has to be examined histopathologically to rule out any neoplastic etiology. We present an adult male with a painful lump over the tibial tuberosity and its management.

## Introduction

Bursae are fluid-filled sacs that are lined by synovium and play their role in protecting tissues like tendons and ligaments from friction against bony surfaces during movements [1]. There are various bursae associated with the knee which are the suprapatellar bursa, prepatellar bursa, infrapatellar bursa, popliteal bursa, pes anserinus bursa, bursa associated with the iliotibial band, lateral collateral ligament and medial collateral ligament of the knee [1]. They have minimal fluid to play their part in reducing friction and protecting tissues. Due to repetitive uses either pertaining to occupation or recreation activities the bursa generates more fluid to counteract the friction resulting in a condition known as bursitis [2]. This resolves over days with conservative treatment but in a handful of cases, this can persist and even lead to ossification or calcification in the substance of the bursa [3,4]. Ossification and calcification (dystrophic or metastatic) in the substance of bursa are rarely described and a detailed workup is necessary before surgical intervention as well as histopathological examination of the excised tissue. In this case report, we present a similar condition wherein the chronic superficial infrapatellar bursitis had ossification in its substance and its management.

## Case presentation

**Figure 1:**
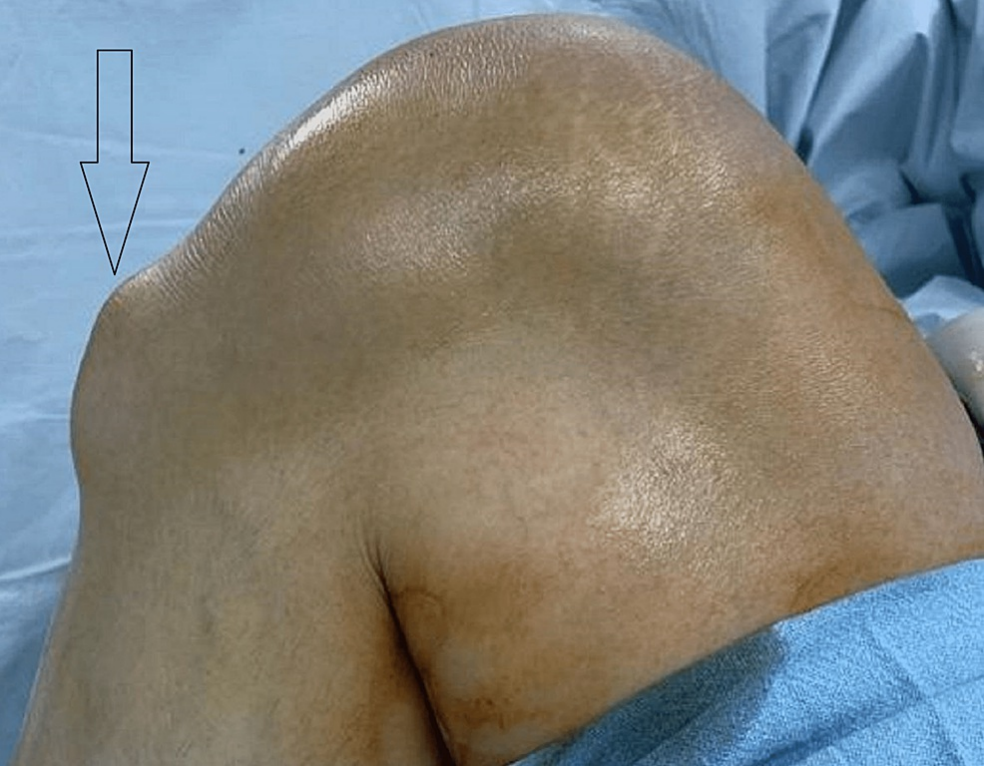
Clinical picture showing lump anterior to the tibial tuberosity

A gentleman of 46 years presented to us with complaints of swelling which was painful to touch over the tibial tuberosity of his right knee. His complaints started two years back insidiously as a small swelling which gradually progressed and attained its present size of 6 cm * 3 cm. The swelling was mobile in both planes and was firm in nature and also tender to touch (Figure 1).

**Figure 2:**
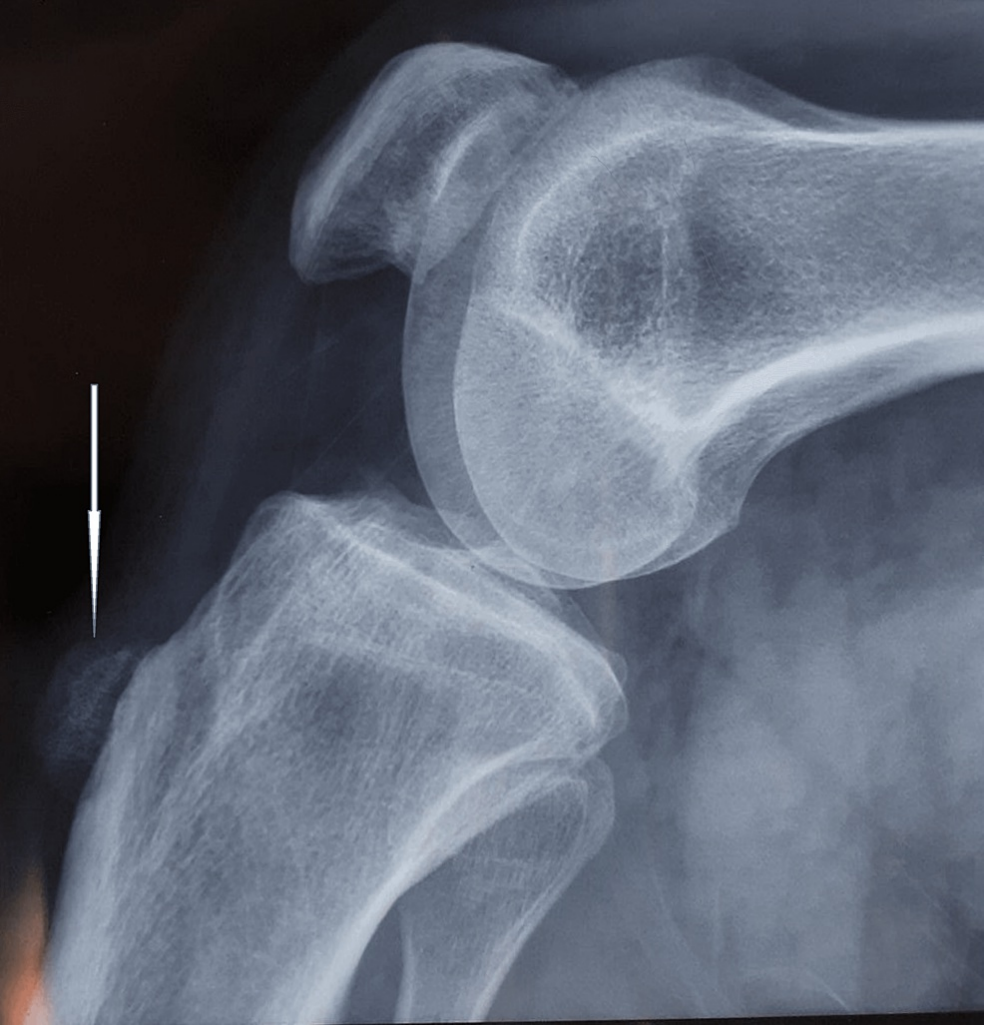
Lateral X-ray demonstrating ossification anterior to the tibial tuberosity

He had difficulty kneeling and squatting because of the swelling. The blood investigations showed normal renal and liver function tests with normal serum calcium and phosphorus (Table 1). The radiographs of the lateral view of the knee were suggestive of ossification anterior to the tibial tuberosity (Figure 2). 

**Table 1:** Blood Investigations

Test	Results	Range
Total Leucocyte Counts (TLC)	4600/cu.microL	4000 – 11000/cu.microL
Differential counts	Neutrophils-71%	Neutrophils 40 – 80%
	Lymphocytes-24%	Lymphocytes 20 -40%
	Monocytes-3%	Monocytes 2-10%
Erythrocyte Sedimentation Rate (ESR)	10 mm/hr	0-15 mm/hr
C-Reactive Protein (CRP)	1.0 mg/dl	0-5 mg/dl
Blood Urea	27 mg/dl	17 – 49 mg/dl
Serum Creatinine	0.6 mg/dl	0.7 – 1.2 mg/dl
Serum Calcium	9.2 mg/dl	8.6 – 10 mg/dl
Serum Phosphorus	3.1 mg/dl	2.5 – 4.5 mg/dl
Alkaline Phosphatase	55 U/L	40 – 129 IU/L

**Figure 3:**
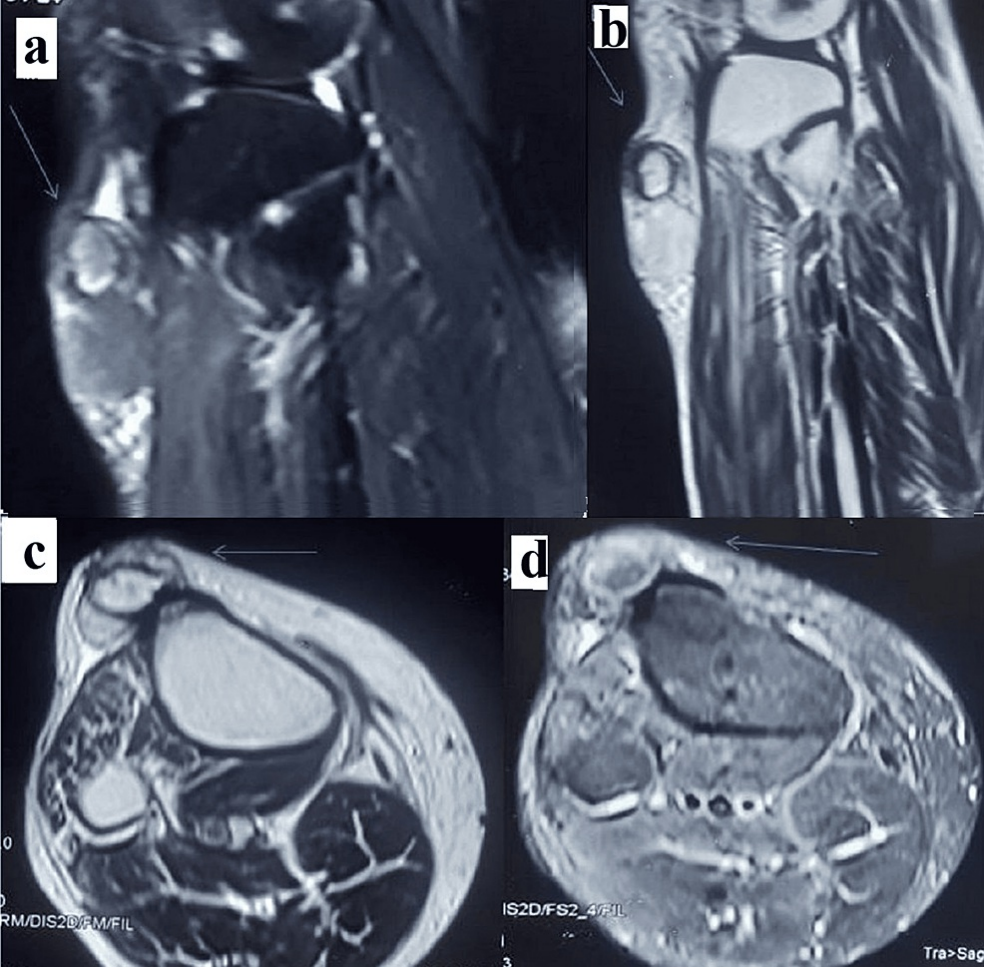
MRI knee MRI knee (with arrows depicting the lesion), (a) is a sagittal T2W image showing a hypo-intense lesion with a hyper-intense rim in the subcutaneous plane anterior to the tibial tuberosity. (b) and (c) are sagittal and axial images T1W images respectively which show hyperintense lesions. The same lesion (d) fat suppression in the STIR sequence suggests chronic ossified infrapatellar bursitis

The MRI images (Figure 3) sagittal and axial images T1W and T2W were suggestive of oval subcutaneous lesion anterior to the tibial tuberosity, which is hyperintense on T1, hypo intense with a hyperintense rim on T2 and show fat suppression suggestive chronic ossified infrapatellar bursitis.

**Figure 4:**
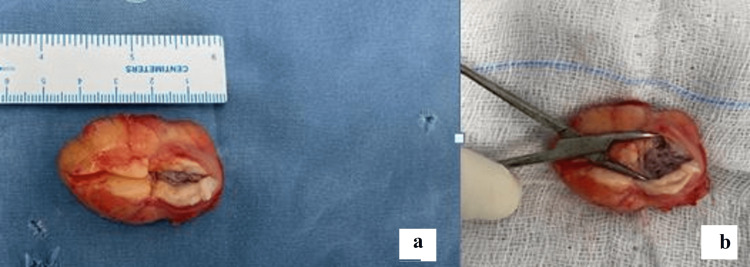
The excised bursal swelling (a) was opened up which revealed a bone-like mass (b) in the center of the swelling with surrounding hypertrophied bursal tissue.

**Figure 5:**
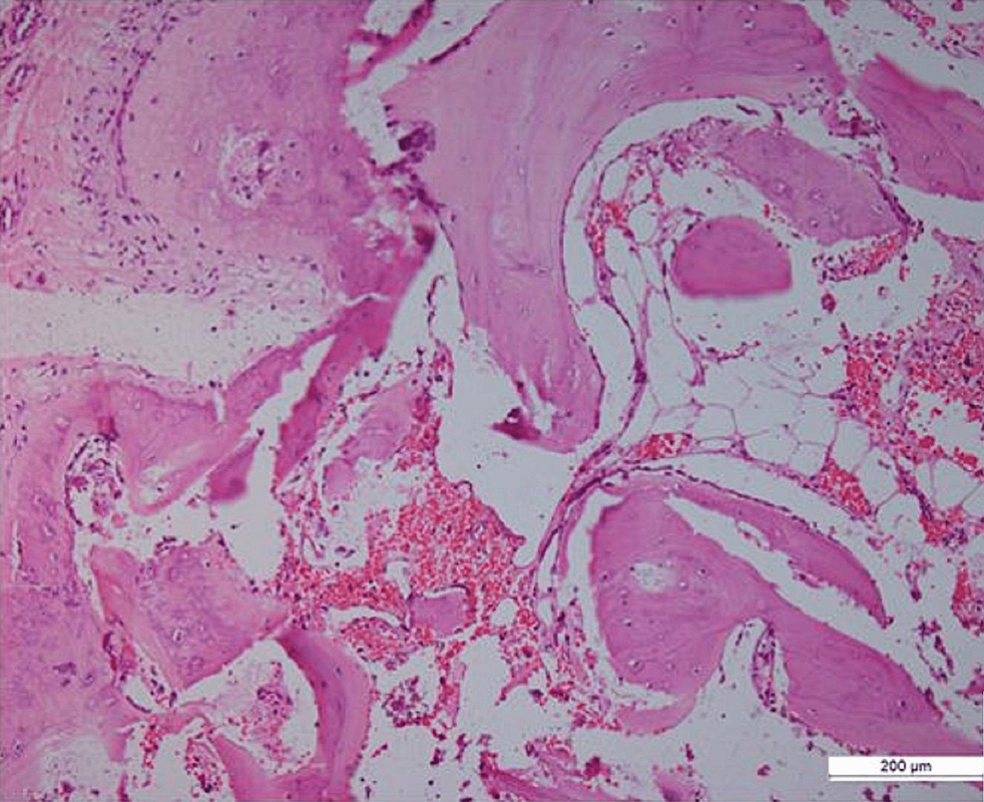
The histopathology pictures showing fibro adipose tissue with multifocal new bone formation and dystrophic calcification were also seen.

He was operated on under regional anesthesia in a supine position with a tourniquet over the thigh. An S-shaped incision was centered on the swelling over the tibial tuberosity and a hard well-defined mass was identified with a few fibers of the patellar tendon attached to it. The mass was removed in total as a single piece and the few fibers of the patellar tendon that were seen attaching to the mass were repaired to the intact tendon. The wound was closed and the patient was discharged the next day. The excised bursal mass was sent for histopathological examination. The mass was cut open which revealed hypertrophied bursal tissue (Figure 4a) and an ossified mass in the center of it (Figure 4b). Histopathological examination was suggestive of fibro adipose tissue with multifocal metaplastic new bone formation and dystrophic calcification also seen (Figure 5).

He was started on full weight bearing and isometric quadriceps exercises in the postoperative period. He followed up at two weeks, suture removal was done and knee range of movement exercises were started. At four months follow up he was able to squat and kneel and had no complaints and attained his full range of movement. At 14 months follow up he had no complaints and there was no recurrence.

## Discussion

Bursitis can be acute or chronic. Acute bursitis can be due to traumatic etiology (hemorrhagic bursitis) or infective etiology (septic bursitis) [2,3]. In chronic bursitis, the fluid increases to augment its increased function which is reflected in doppler as increased vascularity and angiogenesis [2]. Bursitis is initially treated conservatively with rest and anti-inflammatory drugs but it may require aspiration, debridement, or excision when there is suspicion of infection or no relief with conservative treatment [2,3].

Our patient had chronic bursitis involving the superficial infrapatellar bursa also known as a pretibial bursa. The infrapatellar bursa has two parts: one is superficial to the patellar tendon in the subcutaneous plane and one deep to the tendon and anterior to the tuberosity. They both play a role in protecting the tendon during its movement and can develop bursitis due to increased activity [5-7]. Rarely chronic bursitis can have ossification or calcification in its substance. Only a handful of such reports have been described and some of the bursae around the knee involved are prepatellar bursa [5], and superficial infrapatellar bursa [6].

Heterotopic ossification has been described as the formation of bone at extraskeletal sites whereas calcification is the deposition of calcium salts in extraskeletal sites [8]. Calcification can be metastatic or dystrophic. Metastatic calcification is the deposition of calcium in normal tissues due to pre-existing metabolic abnormality whereas in dystrophic calcification the calcium gets deposited in degenerated tissues. The other differentials of soft tissue ossification and calcifications are benign lesions like gout, tumor calcinosis, and venous malformations with phleboliths. The soft tissue ossifications and calcifications can also simulate malignant pathologies like parosteal osteosarcoma, synovial sarcoma, and periosteal chondroma. These pathologies can be differentiated based on the metabolic profile, radiological investigations, and finally confirmed by histopathology [8].

In our patient, the blood workup was within normal limits and not suggestive of any metabolic bone disease. The radiological work including radiographs and MRI was suggestive of an ossified mass in the substance of infrapatellar bursa without any bony scalloping, invasion, or periosteal reactions suggestive of malignant pathologies. This was confirmed by histopathology of the excised tissue which had bone formation in its substance as well as dystrophic calcification. 

## Conclusions

Bursitis though treatable conservatively, very rarely can have ossification and calcification in its substance which requires surgical intervention. The patient should be investigated for any coexisting metabolic problems before proceeding with surgical intervention. The excision biopsy of such a specimen has to be examined histopathologically to rule out any neoplastic etiology. In our patient, it was suggestive of clergyman’s knee, and radiologically showed ossified bursitis. He underwent an excision biopsy of the same and histopathology revealed metaplastic new bone with dystrophic calcification in its substance.
